# The Many Faces of G Protein-Coupled Receptor 143, an Atypical Intracellular Receptor

**DOI:** 10.3389/fmolb.2022.873777

**Published:** 2022-04-12

**Authors:** Beatriz Bueschbell, Prashiela Manga, Anke C. Schiedel

**Affiliations:** ^1^ Department of Pharmaceutical and Medicinal Chemistry, Pharmaceutical Institute, University of Bonn, Bonn, Germany; ^2^ Ronald O. Perelman Department of Dermatology, Grossman School of Medicine, New York University, New York City, NY, United States

**Keywords:** orphan receptors, GPCRs (G protein-coupled receptors), GPR143, ocular albinism type 1 (OA1), melanosome, intracellular GPCR, pigment cells

## Abstract

GPCRs transform extracellular stimuli into a physiological response by activating an intracellular signaling cascade initiated via binding to G proteins. Orphan G protein-coupled receptors (GPCRs) hold the potential to pave the way for development of new, innovative therapeutic strategies. In this review we will introduce G protein-coupled receptor 143 (GPR143), an enigmatic receptor in terms of classification within the GPCR superfamily and localization. GPR143 has not been assigned to any of the GPCR families due to the lack of common structural motifs. Hence we will describe the most important motifs of classes A and B and compare them to the protein sequence of GPR143. While a precise function for the receptor has yet to be determined, the protein is expressed abundantly in pigment producing cells. Many GPR143 mutations cause X-linked Ocular Albinism Type 1 (OA1, Nettleship-Falls OA), which results in hypopigmentation of the eyes and loss of visual acuity due to disrupted visual system development and function. In pigment cells of the skin, loss of functional GPR143 results in abnormally large melanosomes (organelles in which pigment is produced). Studies have shown that the receptor is localized internally, including at the melanosomal membrane, where it may function to regulate melanosome size and/or facilitate protein trafficking to the melanosome through the endolysosomal system. Numerous additional roles have been proposed for GPR143 in determining cancer predisposition, regulation of blood pressure, development of macular degeneration and signaling in the brain, which we will briefly describe as well as potential ligands that have been identified. Furthermore, GPR143 is a promiscuous receptor that has been shown to interact with multiple other melanosomal proteins and GPCRs, which strongly suggests that this orphan receptor is likely involved in many different physiological actions.

## Introduction

G protein-coupled receptor 143 (GPR143) is encoded by the ocular albinism 1 (*OA1*) gene, which was first cloned because of its role in the pathogenesis of ocular albinism, a disorder caused by dysfunction of pigment producing cells (Bassi et al., 1995). Lack of expression of functional GPR143 in pigment producing cells in the skin and hair (known as melanocytes) results in morphologic abnormalities of the organelles in which the pigment melanin is synthesized (known as melanosomes). The effects of pathogenic *OA1* mutations are more consequential in the pigment cells of the eyes (including melanocytes and retinal pigment epithelium/RPE), resulting in severe reduction of visual acuity. Early studies of the protein were thus limited to melanocytes and RPE, however recent investigations have addressed a broader role for GPR143 as one of the few intracellular G protein-coupled receptors (GPCRs).

## G protein-Coupled Receptor Related Structural Elements of GPR143

GPCRs, the largest family of membrane receptors, mediate almost all (patho)physiological functions in mammals (Fredriksson et al., 2003; Rosenbaum et al., 2009). Among the over 800 known GPCR gene sequences, approximately half encode receptors involved in sensory perception. Of the remaining non-sensory receptors, about 100 are orphan GPCRs (oGPCRs) for which either ligands and/or downstream signaling pathways are unknown (Fredriksson et al., 2003; Hauser et al., 2017; Harding et al., 2018). Since GPCRs have been established as a major class of protein that can be targeted by pharmaceuticals, deorphanization of oGPCRs may unlock valuable physiological information and provide new therapeutic approaches to improve human health (Rask-Andersen et al., 2014; Hauser et al., 2017; Jabeen and Ranganathan, 2019). GPR143 is one such oGPCR, an atypical receptor that is localized intracellularly in endolysosomes and melanosomes rather than the cell membrane where most other GPCRs function (O’Donnell et al., 1976; Samaraweera et al., 2001; Schiaffino and Tacchetti, 2005). Aside from assignment to a receptor family, the precise function of GPR143 still remains to be determined.

### GPR143 and Its Relation to the GPCR Superfamily

From a structural perspective, all GPCRs display the same architecture—seven transmembrane alpha helices (TMs) connected by three intracellular and three extracellular loops (ICL1-3, ECL1-3). N-termini and C-termini, which the majority of GPCRs possess, can vary in length and in regulatory roles they play in GPCR signaling (Coleman et al., 2017; Odoemelam et al., 2020).

GPCRs can also be classified into subfamilies based on sequence homology. Vertebrate GPCRs can be subdivided by the GRAFS system (Glutamate, Rhodopsin, Adhesion, Frizzeled/Taste2, Secretin) (Fredriksson et al., 2003; Schiöth and Fredriksson, 2005; Alexander et al., 2017, 2019; Hu et al., 2017). Alternatively, a system of subdivision by functional similarities and sequence homology, which includes non-vertebrate GPCRs, has also been developed. GPCRs can be categorized into six classes by the ABC system: Class A—rhodopsin-like receptors, Class B—secretin family, Class C—metabotropic glutamate receptors, Class D—fungal mating pheromone receptors (non-vertebrate receptors), Class E—cAMP receptors (non-vertebrate receptors) and Class F—frizzled (FZD) and smoothened (SMO) receptors (Attwood and Findlay, 1994; Kolakowski, 1994; Hu et al., 2017). In order to classify a GPCR into a specific class it has to have been shown, by phylogenetic studies, that the candidate shares at least 20% sequence identity in the TMs, indicating likely evolution from a common ancestor (Alexander et al., 2019). A number of receptors, including GPR143, which do not display sufficient homology to known GPCR subfamilies remain to be assigned to a specific class.

**FIGURE 1:**
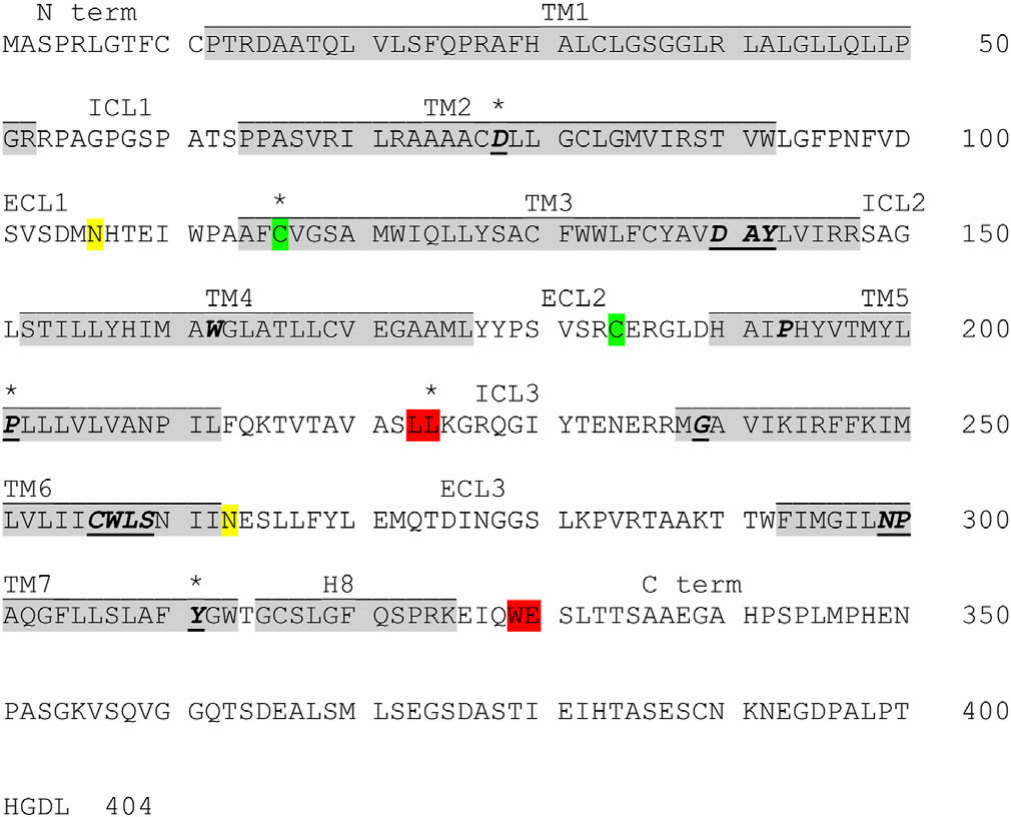
Protein sequence of GPR143 with highlighted features and residues. Grey: transmembrane domains (TM) as predicted by GPCRdb (Isberg et al., 2015, 2016; Kooistra et al., 2021); yellow: potential glycosylation sites N106 in ECL1 and N263 in ECL3 (Samaraweera et al., 2001); green: highly conserved cysteine residue in TM3 (C3.25) which might form a disulfide bond with C184 in extracellular loop 2 (ECL2); red: sorting signals for intracellular localization (dileucine motif (L223/L224) in the intracellular loop 3 (ICL3) and tryptophan-glutamic acid doublet (W329/E330) in the C terminal tail) (Piccirillo et al., 2006). Conserved residues found in class A GPCRs or similar motifs are bold, italic and underlined, motifs common in class B GPCRs are bold and italic, residues known to cause ocular albinism type 1 when mutated are marked with an asterix (for references see main text).

Apart from sequence identity, the growing number of structure-function studies and resolved crystal structures have revealed that there are common structural and functional motifs which are crucial for the activation of each respective GPCR class (Latek et al., 2013; Moreira, 2014; Isberg et al., 2015; Zhou et al., 2019). In order to easily localize such motifs and to compare them to other GPCR classes, all GPCR residues can be annotated using the Ballesteros and Weinstein nomenclature (Ballesteros and Weinstein, 1995). Based on this nomenclature, the first digit identifies the TM helix and the second digit the residue position in relation to the most conserved residue of each TM helix (assigned index number 50). Numbers decrease towards the N-terminus and increase towards the C-terminus (Ballesteros and Weinstein, 1995; Moreira, 2014). We will briefly discuss the most prominent structural and functional motifs of class A and B receptors, which may be relevant for GPR143. Aside from class D and E receptors, which are only present in invertebrates, classes C and F do not display any similarities with GPR143. In addition we will highlight other residues, such as glycosylation sites and sorting signals. These key residues and features are annotated in Figure 1.

### Key Residues Relevant for GPR143

GPR143, the protein product of the *OA1* gene, was found to span the plasma membrane seven times, indicating that it could be a potential GPCR as far back as 2007 when the 404 amino acid length protein, was classified as orphan receptor GPR143 (Sone and Orlow, 2007). Here we summarize the key residues found in class A and B receptor families and compare them to GPR143, as early studies by Schiaffino *et al.* demonstrated that the receptor shows weak similarities with classes A and B (Schiaffino et al., 1999) (see Figure 1). Similar findings were described by Gosh et al. who investigated GPR143 using a 3-dimensional modeling approach (Gosh at al., 2012). In the IUPHAR guide to pharmacology, GPR143 is still listed in the family of “other 7 TM proteins” (Alexander at al., 2019; http://www.guidetopharmacology.org/GRAC/ObjectDisplayForward?objectId=203, accessed on 9 February 2022) and in the GPCRdb it is listed among “other GPCR orphans” (Kooistra et al., 2021).

The structural and functional motifs that define class A are the most extensively studied with relation to their activation mechanism. Class A GPCRs, also referred to as rhodopsin receptors, include many functionally diverse receptor members. Diverse physiological actions such as inter- and intracellular communication, sense of sight, smell taste and touch, chemotaxis and neurotransmission are mediated through class A GPCRs (Fredriksson et al., 2003; Mombaerts, 2004; Moreira et al., 2010; Moreira, 2014; Isberg et al., 2016). According to Zhou *et al.* (Zhou et al., 2019) and numerous other studies (for example, (Moreira, 2014; Filipek, 2019), the most important (and conserved) motifs are:

1) The interaction of the cytoplasmic “ionic lock” in TM3, which is disrupted when the receptor is activated with the consensus “(D/E)R (Y/M)” (3.49–3.51) with D/E (6.30) in TM6 (Ballesteros et al., 1998, 2001; Schneider et al., 2010; Jacobson et al., 2014; Yuan et al., 2014; Feng et al., 2017; Roth et al., 2017; Schönegge et al., 2017; Alhadeff et al., 2018); the characteristic DRY motif in TM3 is DAY in GPR143 (Ghosh et al., 2012), while the counterpart in TM6 D/E 6.30 is G in GPR143.
2) The hydrophobic arginine cage around the conserved arginine (R3.50) of the DRY motif, which restrains its conformation in the inactive state of the receptor consisting of two hydrophobic amino acids (such as L, V, I or M) in TM3 and TM6 (3.46, 6.37) (Ballesteros et al., 1998; Visiers et al., 2002; Weinstein, 2006; Caltabiano et al., 2013); the arginine cage is not present in GPR143.
3) The NPxxYxF motif in TM7, which forms the interaction of a tyrosine (7.53) in TM7 with the phenylalanine (7.60) in HX8 together with the side chain and backbone of an arginine in TM2 (2.40) through binding of a water molecule (Prioleau et al., 2002; Fritze et al., 2003; Angel et al., 2009; Hofmann et al., 2009; Rasmussen et al., 2011; Trzaskowski et al., 2012; Venkatakrishnan et al., 2016; Schönegge et al., 2017; Filipek, 2019); this motif is partially found in GPR143 represented by N7.41and P7.42 (Ghosh et al., 2012), however the tyrosine in the motif is missing in GPR143, only the conserved Y7.53 is found.
4) A coordinated conformational change upon ligand coupling of aromatic residues in TM6, also known as CWxP motif around a very conserved tryptophan (6.48) that leads to disruption of the ionic lock and outward movement of TM6 (called Rotamer Toggle Switch), while TM7 undergoes inward movement towards TM5 (Weinstein, 2006; Hofmann et al., 2009; Nygaard et al., 2009; Holst et al., 2010; Standfuss et al., 2011; Trzaskowski et al., 2012; Valentin-Hansen et al., 2012; Tehan et al., 2014; Zhang et al., 2015; Venkatakrishnan et al., 2016; Plazinska et al., 2017; Eddy et al., 2018; Kaiser et al., 2018; Filipek, 2019); instead of CWxP, CWLS is found in GPR143 in the same position (Ghosh et al., 2012).
5) The PIF motif comprising residues P5.50, I3.40 and F6.44 (Ballesteros and Weinstein, 1995; Ishchenko et al., 2017; Schönegge et al., 2017; Kato et al., 2019; Hilger, 2021; Smith, 2021); the PIF motif is also not present in GPR143, only P5.50 itself is found.
6) The Na^+^-pocket at a conserved aspartic acid (2.50) (Liu et al., 2012; Yuan et al., 2013; Zhang et al., 2013; Katritch et al., 2014; Eddy et al., 2018; Vickery et al., 2018; White et al., 2018; Ye et al., 2018; Chen S. et al., 2019; Filipek, 2019; Agasid et al., 2021); D2.50 important for Na^+^ binding is also present in GPR143.

Some of the conserved residues in the helix packing clusters of class A GPCRs are also found in GPR143 (G/S1.46, A2.47, A3.38, S/A4.53, P5.50), however GPR143 does not contain all of the described switches (Sanchez-Reyes et al., 2017). The conserved disulfide bond between C3.25 on TM3 and C184 (ECL2), common in most GPCRs, is also found in the GPR143 sequence (Venkatakrishnan et al., 2013), see Figure 1.

Adhesion and secretin receptors were initially classified as two distinct families, however the secretin GPCRs were later shown to have some structural resemblance to adhesion GPCRs and through phylogenetic analysis it was determined that secretin GPCRs evolved from adhesion GPCRs (Nordström et al., 2009; Hamann et al., 2015). The secretin receptor family was labeled class B1 and the adhesion receptor family class B2 (Pándy-Szekeres et al., 2018; Scholz et al., 2019).

Distinct from class A receptors, secretin receptors possess a very large N-terminus, which is also called extracellular domain (ECD) that is important for ligand recognition and activation of the receptor together with the TM domains (Hoare, 2005; Parthier et al., 2009; Pal et al., 2012; Ramos-Álvarez et al., 2015; Karageorgos et al., 2018). The ECLs and the upper regions of the TMs form the so-called “J domain” of class B receptors (Karageorgos et al., 2018). GPR143 does not have an ECD, however it may have been lost in evolution, since remnants of at least one former *OA1* exon is still found on the Y chromosome (Van Laere et al., 2008; Pandey et al., 2013) (see chapter “Evolutionary Aspects”).

Only a few studies have attempted to investigate adhesion receptors from a pharmacological perspective, such as through mutational studies (Peeters et al., 2016; Nazarko et al., 2018; Arimont et al., 2019; Beliu et al., 2021). From these studies important residues and from sequence alignments with sequences from class A and B1 relevant residues in class B2 receptors have been identified (Nijmeijer et al., 2016; Arimont et al., 2019). For instance, most class B1 conserved residues are also present in class B2 such as S1.50 (1.46 in class A), H2.50 (2.43 in class B1), E3.50 (3.46 in class A), W4.50 (4.50 in class A), N5.50 (5.54 in class A), G6.50 (6.45 in class A) and G7.50 (7.46 in class A) (Wootten et al., 2013; Nijmeijer et al., 2016; Arimont et al., 2019). Furthermore, a conserved proline residue of TM5 of secretin receptors P5.42 can also be found in adhesion receptors as well as a conserved tryptophan at position 6.53 which is similar to the conserved tryptophan at position 6.48 in class A GPCRs which is part of the CWxP motif and may act as potential toggle switch as seen for class A (Nijmeijer et al., 2016; Plazinska et al., 2017; Ping et al., 2021). The conserved P5.42 is one of the few residues GPR143 has in common with class B receptors as well as the highly conserved W4.50 found in class A and B.

### Other Structural Features

GPR143 possesses two distinct sorting signals for lysosomal and melanosomal localization, a dileucine motif in ICL3 and a tryptophan-glutamic acid doublet in the C terminal tail. Both are essential and sufficient for localization to organelles (Piccirillo et al., 2006). When mutated to alanine residues, the receptor is primarily localized to the plasma membrane (Piccirillo et al., 2006; De Filippo et al., 2017b). In addition to the sorting signals, Giordano et al. demonstrated that GPR143 intracellular sorting and ubiquitination are dependent upon functional components of endosomal sorting complexes required for transport (ESCRT) complex (Giordano et al., 2011). Intracellular sorting and down-regulation were also associated with the amount of ESCRT-0, -I, and -III subunits, as their depletion or overexpression inhibited GPR143 degradation together with retention in the endosomal system (Giordano et al., 2011). Giordano and co-workers hypothesized, based on their results, that the ESCRT machinery ubiquitinates GPR143 in intralumenal vesicles of multivesicular endosomes thereby allowing for modulation between downregulation and GPR143 delivery to melanosomes (Giordano et al., 2011).

The receptor also contains two potential glycosylation sites, one in ECL1 (N106) and the second in ECL3, however evidence for glycosylation has only been shown for N106 (Samaraweera et al., 2001) (see Figure 1).

### Evolutionary Aspects

In the gene (NG_009074.1) and nucleotide (NM_000273.3) databases GPR143 is referenced with 9 exons, of which 7 contain coding sequence regions and the protein is 404 amino acids long (NP_000264.2; UniProt ID P51810). Several shorter isoforms of the protein have been predicted, e.g., isoform X1 (XP_005274598.1) with 386 amino acids and isoform X2 (XP_024308155.1) with 320 amino acids.

The *OA1* gene is located on chromosome Xp22.2. Adjacent to GPR143, at the same chromosomal location, is SHROOM2, which is also highly expressed in the retina and may also be associated with ocular defects, similar to GPR143 (Van Laere et al., 2008; Pandey et al., 2013). Interestingly, it may contain one or more non-coding GPR143 exons which were lost during evolution. Mammalian sex chromosomes evolved from homologous autosomes after a series of recombination suppression and inversion events that rendered the Y chromosome relatively short. So-called “small pseudoautosomal regions” remain on the Y chromosome and can be mapped to distinct evolutionary strata (Van Laere et al., 2008; Pandey et al., 2013). Pseudogenes of the SHROOM/GPR143 gene cluster are still present on the Y chromosome (Van Laere et al., 2008).

## GPR143 Function

GPCRs typically transform extracellular stimuli into a physiological response by activating an intracellular signaling cascade initiated by binding to G proteins. Regarding GPR143, little is known about its intracellular signaling or its binding of ligands, most probably due to its unique localization within the cell. However, a few studies have identified potential ligands for GPR143. The topological orientation of the receptor suggests that ligands would have to bind from the organelle lumen (Sone and Orlow, 2007; Schiaffino, 2010).

### Potential GPR143 Ligands

L-3,4-dihydroxyphenylalanine (L-DOPA) and dopamine have been proposed as ligands by Lopez et al. in 2008 (Lopez et al., 2008). While L-DOPA is an intermediate compound in the biosynthesis of melanin and the conversion of L-tyrosine to L-DOPA the rate-limiting step of the process (D’Mello et al., 2016), dopamine is not part of the synthesis but can be produced through hydroxylation of L-DOPA (Daubner et al., 2011). In addition, Staleva and Orlow suggested, based on their results in a yeast-based study, that activating compounds or proteins for GPR143 may be located in the 100,000 g fraction of cultured, heavily pigmented melanocytes, which contains melanosomes (Staleva and Orlow, 2006).

In radioligand-binding experiments an equilibrium dissociation constant (Kd) of 9.35 µM for L-Dopa and a Kd of 2.39 µM for dopamine were obtained (Lopez et al., 2008). By measuring the intracellular calcium release through G_q/11_ protein coupling in CHO cells, only L-DOPA was shown to activate GPR143, while dopamine displayed antagonist properties instead. After treatment with L-DOPA GPR143 and β-Arrestin colocalized at the plasma membrane, indicating that GPR143 is able to change its localization in a ligand-dependent manner (Lopez et al., 2008). In another study by Hiroshima et al., a Kd of 79.1 µM was measured for L-DOPA using murine GPR143, but only in RPE-derived cells, while no calcium release was detected in CHO cells. The authors further reported that the binding of L-DOPA to murine GPR143 was competitively antagonized by L-DOPA cyclohexyl ester (DOPA-CHE) (Hiroshima et al., 2014). Since the discovery of L-DOPA as potential ligand for GPR143, many studies have been conducted based on this finding, for example clinical studies to evaluate L-DOPA as a therapy for ocular albinism, which were not conclusive (Summers et al., 2014). Although it is most likely that GPR143 is a receptor for L-DOPA, there are also contradictory findings (see below).

Goshima and co-workers, who performed microinjections into the nucleus tractus solitarius (NTS) to investigate cardiovascular actions of L-DOPA through GPR143, could not exclude that dopamine converted from L-DOPA in non-neuronal tissues would be also able to exert cardiovascular and/or renal actions through β-adrenergic receptor and dopamine receptors D_1_R and D_2_R (Goshima et al., 2014). It was shown that D_1_R activity in vascular smooth muscle is associated with vasodilatation (Kohli, 1990). Moreover D_2_R were shown to exert their effects on sympathetic nerve terminals which resulted in a decrease of noradrenaline release (Dubocovich and Langer, 1980). Also effects on renal blood flow, glomerular filtration rate, urinary sodium, and water excretion as well as promotion of phosphate excretion and antagonizing the hydro-osmotic effect of vasopressin were described (Goshima et al., 2014).

In addition, a study by Ueda *et al.* showed that L-DOPA (10–100 mg/kg, i.p.) induced ptosis in wildtype (WT) and GPR143-knockout (KO) mice, which were pretreated with 3-hydroxybenzylhydrazine, a central aromatic L-amino acid decarboxylase inhibitor, used to prevent conversion of L-DOPA to dopamine (Ueda et al., 2016; Goshima et al., 2019a). Ptosis, which is also called “lazy eye,” is described as the falling or dropping of the upper eyelid (Finsterer, 2003). Similar malfunctioning of the motoric system (“motor blocks”) can sometimes result from either short- or long-term L-DOPA treatment of Parkinson’s disease (Giladi et al., 1992; Dewey and Maraganore, 1994; Goshima et al., 2019a). Since L-DOPA induced ptosis in both WT and GPR143-KO mice, the authors suggest that there may be GPR143-dependent and independent mechanisms for inducing ptosis (Ueda et al., 2016; Goshima et al., 2019a).

In 2017 De Filippo et al. used a high throughput screening (HTS) approach to identify new pharmacological tools for further investigation of GPR143 signaling (De Filippo et al., 2017a). Due to the intracellular localization of GPR143 a mutant was created, which localized to the plasma membrane (Piccirillo et al., 2006), in order to ensure that concerns about test compounds permeating into the cells and reaching the receptor were addressed. The β-Arrestin recruitment assay was chosen as a suitable assay for HTS screening since GPR143 showed high constitutive activity, while the melanin assay was chosen to validate potential candidates. Pimozide, niclosamide and ethacridine lactate were identified as new inverse agonists for GPR143, however are yet to be independently confirmed. Pimozide is a dopamine receptor D_2_R and D_3_R-antagonist (Schwinn et al., 1976; Sokoloff et al., 1992), niclosamide was described as anthelmintic teniacide with antitumor activity (Pan et al., 2012) and ethacridine lactate as an antiseptic. In the same study by De Filippo et al. no Ca^2+^ response following L-DOPA stimulation could be observed in CHO cells, which is in contradiction to the findings by Lopez et al. (Lopez et al., 2008; De Filippo et al., 2017a). To further characterize GPR143 and understand its actions there is a need for the discovery of new, highly potent compounds as pharmacological tools.

### GPR143 Signaling Pathways

Schiaffino et al. showed that GPR143 associates with several G_α_ subunits and G_β_ (Schiaffino et al., 1999; Schiaffino and Tacchetti, 2005). Furthermore, Innamorati et al. discovered that GPR143 also associates with β-Arrestin, even in the absence of a ligand (Innamorati et al., 2006). This finding was further confirmed by De Filippo et al. who also showed ligand-independent β-Arrestin recruitment in an *in vitro* assay system (De Filippo et al., 2017a). Moreover, a study by Lopez et al. who also discovered L-DOPA as potential ligand for GPR143, observed an influx of intracellular calcium and recruitment of β-Arrestin, when cells were treated with L-DOPA at high concentrations (Lopez et al., 2008). McKay et al. showed that myocilin plays a role in ligand-dependent β-Arrestin-recruitment to GPR143, which leads to endocytosis (McKay et al., 2013).

In 2008, a study by Young et al. investigated the relationship between GPR143 and the G_αi_ family comprising the closely-related members, G_αi1_, G_αi2_, and G_αi3_ (Young et al., 2008). The G_αi_ proteins also locate to intracellular membranes including Golgi and endosomal membranes (Gohla et al., 2007; Young et al., 2008) and were found to be associated with membrane trafficking and fusion events (Young et al., 2008). In addition, G_αi3_ is expressed in fetal and adult human RPE cells and the inner neural retina (Jiang et al., 1991; Oguni et al., 1996; Young et al., 2008). By studying the RPE density and morphology of melanosomes of G_αi3_^−/−^ and GP143^−/−^ knockout mice, the authors were able to observe the same phenotype in both mice genotypes (Young et al., 2008). Furthermore Young and co-workers investigated routing of the optic tract, which is established during development and disrupted in ocular albinism. Both G_αi3_^−/−^ and GP143^−/−^ knockout mice were found to have the same optic nerve misrouting, which suggests that GPR143 signaling may be executed through G_αi3_ (Young et al., 2008). Hence, it was suggested that a potential GPR143-G_αi3_ signaling cascade is involved in the correct routing of axons through the optic chiasm and that G_αi3_ is part of the same signal transduction pathway as GPR143, which also regulates melanosome biogenesis (Young et al., 2008). Besides G_ai3_, also G_o_ and G_q_ were suggested as potential G proteins partners for GPR143 (Young et al., 2008).

GPR143 may also play a role in melanomagenesis and melanoma progression. Stimulation of the RAS/RAF/mitogen activated protein kinase (MEK)/extracellular signal-regulated kinase (ERK) pathway by the Epidermal Growth Factor (EGF) and Platelet Derived Growth Factor (PDGF), which play a role in proliferation and survival of tumor cells, resulted in enhanced GPR143 expression and migration of the melanoma cells (Bai et al., 2014).

### Expression and Localization of GPR143

GPR143 is most abundantly expressed in pigment producing cells in the skin and eyes. The pigment produced by these cells is melanin, which plays a central role in protecting human skin and eyes from the deleterious effects of ultraviolet light (UV). Pigment cells and melanin are also required for normal development of the optic system and function of the eye (Williams, 2018). Pigment cells in the skin and eyes are continually subjected to environmental stimuli including UV, visible light and chemotoxins. These stimuli can instigate cellular changes that lead to tumorigenesis (Gilchrest et al., 1999) and age-related macular degeneration (Xia et al., 2019).

Epidermal melanocytes produce melanin in the skin and hair, and RPE in the eye. RPE are required for photoreceptor function and formation of the blood–retinal barrier (Strauss, 2005). Melanocytes are also found in mucosal tissues, the inner ear, eyes and brain (Yamaguchi and Hearing, 2014).

Melanin is produced in membrane-bound organelles called melanosomes. Melanosome biogenesis is a *de novo* process involving four stages of maturation (Seiji et al., 1963). Melanosomes originate from endolysosomal structures (Raposo et al., 2001) and share a lineage with lysosomes (Orlow, 1995). In melanocytes, GPR143 localizes to the melanosomal membrane. When expressed exogenously in non-pigmented cells GPR143 localizes to lysosomes, e.g. in CHO, COS7 or HeLa cells (Schiaffino et al., 1999; Shen et al., 2001; Piccirillo et al., 2006; De Filippo et al., 2017b). However, the intracellular localization was questioned by McKay et al. in a commentary in relation to the paper by De Filippo et al. (De Filippo et al., 2017b; McKay et al., 2017). McKay at al. claimed that intracellular localization was only due to tyrosine present in culture media and that lack of Ca^2+^ response was also due to presence of tyrosine which acts as a weak antagonist (McKay et al., 2017). De Filippo et al. presented, in an author response, further data that supported their finding of intracellular localization even in tyrosine-free medium, as well as lack of Ca^2+^ response in medium without tyrosine (De Filippo et al., 2017c). Intracellular localization in heterologous systems was also shown by other groups as stated above (Schiaffino et al., 1999; Shen et al., 2001; Piccirillo et al., 2006).

Aside from the lack of defined function for GPR143, the expression profile of the receptor in different tissues, apart from the skin and eyes, is also poorly delineated. Fukuda et al. showed in mice, GPR143 is highly expressed in several regions of the brain including pyramidal neurons in the cerebral cortex (Fukuda et al., 2015). By using RT-PCR, GPR143 mRNA was detected in the central nervous system, olfactory bulb, corpus striatum, hypothalamus, hippocampus, midbrain, cerebellum and lower brain stem. While the highest expression was observed in the cerebral cortex and hypothalamus; moderate expression was shown in the olfactory bulb, hippocampus, midbrain and lower brain stem and lastly, low expression was seen in the corpus striatum and cerebellum (Fukuda et al., 2015). The receptor was also found to be expressed in the habenular nucleus, substantia nigra, medulla oblongata and NTS (Fukuda et al., 2015). In addition, Masukawa et al. performed an immunohistochemical analysis of GPR143 expression in the adult rat brain (Masukawa et al., 2014). GPR143-immunoreactive cells were found in the hippocampus, cerebral cortex, cerebellum cortex, striatum, substantia nigra, hypothalamic median eminence and supraoptic nucleus, NTS and caudal ventrolateral medulla and rostral ventrolateral medulla, medial habenular nucleus and olfactory bulb. Outside the brain GPR143 was found in the lungs, heart, kidneys, spleen and liver. The expression of the receptor in the kidney was most abundant and comparable to expression in the cerebral cortex and hypothalamus (Fukuda et al., 2015).

At the cellular level, GPR143 was found in cell bodies of pyramidal neurons, also excitatory neurons and various nuclei such as the NTS can express the receptor. Regarding non-neuronal expression of GPR143, Fukuda et al. discovered GPR143-positive cells in the convoluted tubules in the kidney, in the splenic capsule and red pulp of the spleen, hepatocytes around the hepatic vein of the liver, alveolar epithelial cells and bronchial tubes of the lung, smooth muscle cells around the respiratory bronchiole as well as in the basal lamina and connective tissues in their immunostaining experiments. Interestingly, expression of GPR143 throughout the body overlaps, in most parts, with the expression pattern of dopamine, angiotensin and adrenergic receptors, which are all involved in sympathetic signaling (Fukuda et al., 2015).

## GPR143 and Diseases

### GPR143 Role in Melanocytes and Ocular Albinism

Albinism refers to a group of genetic conditions associated with hypopigmentation (Bassi et al., 1995). The disorder can affect the skin, hair and eyes (oculocutaneous albinism) or only the eyes (ocular albinism). Several forms, defined by the mutated gene, exist, among them Ocular Albinism Type 1 (OA1) (Bassi et al., 1995; Shen et al., 2001). OA1, also called X-linked ocular albinism of the Nettleship-Falls type has a prevalence of 1–9/1 000 000 and an estimated birth prevalence of 1/60 000 to 1/150 000 live male births (https://www.orpha.net/consor/cgi-bin/OC_Exp.php?Expert=54&lng=EN, accessed 3 January 2022). Since OA1 is inherited in an recessive X-linked manner it affects mostly males. Heterozygous females can manifest a less severe phenotype (Falls, 1951; Lang et al., 1990; Preising et al., 2001; Shen et al., 2001). The OA1 phenotype is defined by significant reduction of visual acuity, nystagmus, strabismus, marked photophobia and loss of stereoscopic vision due to misrouting of the optic tract (Creel et al., 1990; Kriss et al., 1992; Bassi et al., 1995). In addition, iris translucency, foveal hypoplasia and hypopigmentation of the retina (O’Donnell et al., 1976; Bassi et al., 1995) is evident upon ophthalmologic examination of OA1 patients. Nystagmus, which is observed at around 6 months after birth, can be the first indication of ocular albinism (Liu et al., 2007; Mao et al., 2021). Female carriers display a mosaic pattern of ocular (hypo)pigmentation as a result of the inactivation of the affected X chromosome (Falls, 1951; Lang et al., 1990).

At the cellular level, the most prominent feature of OA1 is the formation of macromelanosomes (O’Donnell et al., 1976; Garner and Jay, 1980; Wong et al., 1983; Yoshiike et al., 1985). Consequently, OA1 can be seen as a systemic disorder affecting the biogenesis of melanosomes (Traboulsi, 2012). Despite affecting melanosome morphology, effects of *OA1* mutations on the skin appear to be mild (Bassi et al., 1995).

**FIGURE 2:**
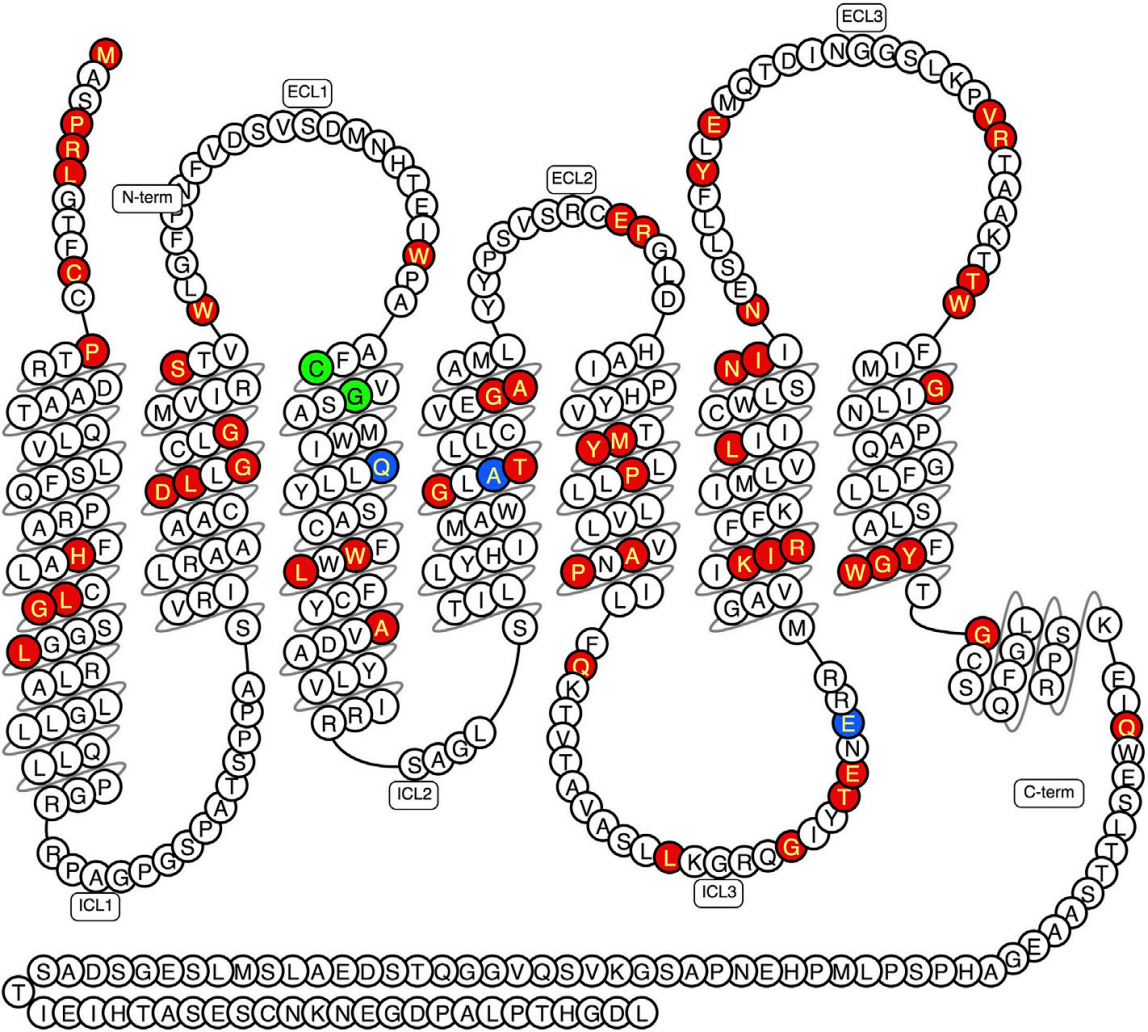
Protein sequence of GPR143 showing pathogenetic point mutations which lead to the OA1 phenotype (red) and mutations which cause a mild variant of OA1 (blue). For some mutated amino acids, either strong or weak OA1 phenotypes were reported (green). The snake plot was created using GPCRdb (Kooistra et al., 2021).

Mutations or loss of function (LoF) in the *OA1* gene (GPR143) results in OA1 (Shen et al., 2001). Moreover, the severity of OA1 is associated with the degree of skin pigmentation, with symptoms more severe in individuals whose skin are lightly pigmented and less severe in individuals with highly pigmented skin (O’Donnell, 1978; Shiono et al., 1995). The incidence of ocular albinism also varies between populations (Zhu et al., 2018). To date, over 300 *OA1* point mutations have been identified and 60 of them were reported to result in OA1 (Figure 2 and Supplementary SI, Supplementary Table S1). While most mutations lead to the characteristic OA1-phenotype (red), three residues were identified which, when mutated, lead to a mild form of OA1 (blue). Furthermore, two residues associated with both mild and severe phenotypes were described (green) (Figure 2). Aside from point mutations several pathogenic splicing mutations, small insertions and deletions as well as the deletion of entire exons and other complex mutations were reported (Supplementary Tables S2–6). We collected over 500 mutations for GPR143 taken from literature, the gnomAD_v2.1.1 database (Karczewski et al., 2020), the Albinism database (http://www.ifpcs.org/albinism/oa1mut.html, accessed 12.01.2022), and the Human Gene Mutation Database (http://www.hgmd.cf.ac.uk/ac/index.php, accessed 01.02.2022), however it should be noted that the functional effects of the mutants is unknown (or benign) and presumably most exist in the germline of normal populations.

**FIGURE 3:**
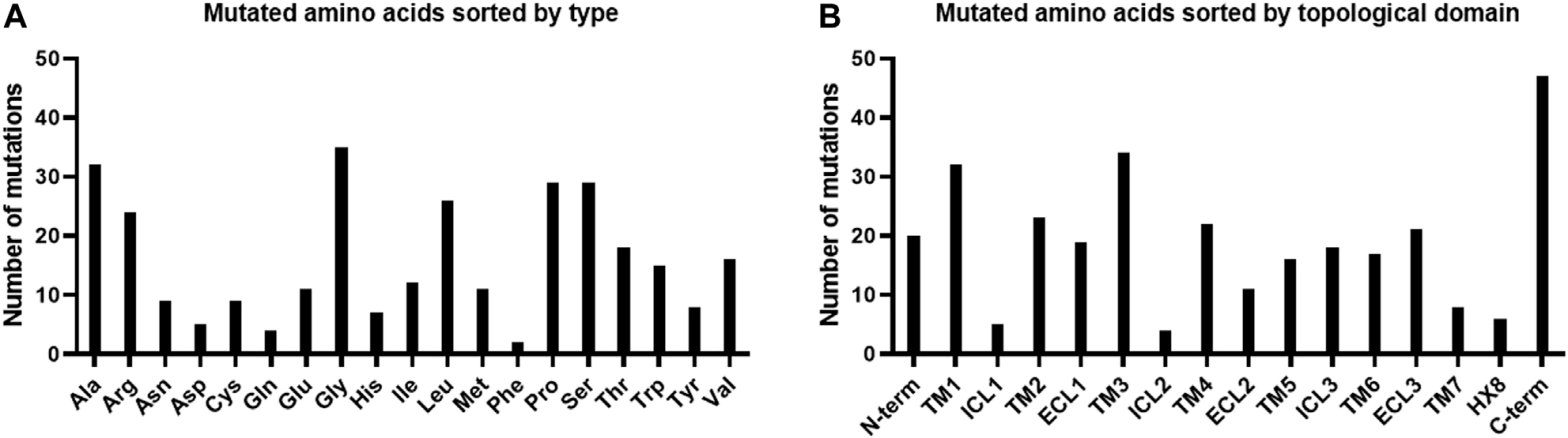
Mutated amino acids affected by point mutations for G protein-coupled receptor 143 sorted by type **(A)** and sorted by topological domain **(B)**.

Regarding the point mutations we identified six specific residues which were found to be mutated more than twice: P1.20/P4 (4x), G2.53/G81 (3x), G2.56/G84 (3x), C3.25/C116 (4x), G3.27/G118 (4x), and W292 (ECL3) (5x). Moreover, when comparing the types of mutated residues we found that glycine and alanine residues were mutated more than 30 times, while phenylalanine was only mutated twice (Figure 3A). When mutated amino acids were sorted by topological domains it becomes obvious that most mutations occur in the C-terminus, while the least frequently mutated ones were found in the ICL1 and ICL2 as well as in the proposed HX8 region (Figure 3B). When comparing the number of mutations within TMs, particularly TM1 and TM3 were most likely to comprise mutated residues, while residues in TM7 were the least affected by mutations (Figure 3B). Some of the mutated residues are highly conserved residues, such as D2.50, C3.25, P5.50 (Figure 1, residues labeled with an asterisk).

Mutations in the genes coding for GPR143 and FERM domain-containing 7 (*FRMD7*), both found on the X-chromosome, have been linked to congenital nystagmus, one of features of ocular albinism that can occur as a non-syndromic condition (Han et al., 2015; Michaud et al., 2019; Wang et al., 2021).

### GPR143 and Cardiovascular Actions

GPR143 has recently been shown to play a role in cardiovascular function through the (NTS) (Goshima et al., 2014; 2019a). The NTS is a sensory nucleus embedded in the medulla oblongata (Šimić, 2019) that receives signals from the solitary tract including facial, taste, gastrointestinal and cardio-respiratory signals (Geerling and Loewy, 2006; Michael Conn, 2008; Zoccal et al., 2014; Šimić, 2019; Martinez and Kline, 2021). Studies by Hiroshima et al*.* suggest that GPR143 serves as an L-DOPA receptor for cardiac response in the NTS. When GPR143 expression was repressed in the NTS, depressor and bradycardic responses to L-DOPA were suppressed (Hiroshima et al., 2014). They further demonstrated that L-DOPA, but not dopamine, produced these cardiovascular effects (Yue et al., 1994; Goshima et al., 2014; Hiroshima et al., 2014).

Masukawa et al. proposed a fine-tuning system of noradrenaline signaling for the regulation of blood pressure through L-DOPA possibly mediated by GPR143. Disruption of this regulation might lead to a number of effects including hypertension, arrhythmia, and other cardiovascular diseases (Masukawa et al., 2017). In addition, GPR143 may also interact with the α_1b_-adrenoceptor. *In vitro* studies using HEK293 cells co-expressing the two proteins confirmed that interaction between GPR143 and α_1b_-adrenoceptor was enhanced by L-DOPA (10–20 nM) treatment in immunoprecipitation assays (Masukawa et al., 2017). They further showed in Förster Resonance Energy Transfer (FRET) and *in situ* proximity ligation assay experiments that GPR143 and α_1b_-adrenoceptor form functional heteromers and that GPR143 has a higher affinity for L-DOPA in the heteromer as compared to the monomeric form (Masukawa et al., 2017). Nakano et al. suggested that α_1b_-adrenoceptor and GPR143 may play a role in the pathology of pulmonary hypertension (Nakano et al., 2022).

### GPR143 in the Central Nervous System

GPR143 is widely expressed in neurons in several regions of the brain (Masukawa et al., 2014; Fukuda et al., 2015) and may play a role in the etiology of Parkinson’s disease (PD). L-DOPA is the most effective therapeutic for PD. It was developed as an inactive precursor for dopamine, which would be delivered to the brain to compensate for dopamine deficiency (Hornykiewicz, 2006; Goshima et al., 2019a). L-DOPA is then converted to dopamine in the brain by aromatic L-amino acid decarboxylase and alleviates motoric symptoms of PD such as akinesia, tremor, rigidity and bradykinesia (Xia and Mao, 2012; Moustafa et al., 2016).

Goshima et al., and others, have presented evidence that L-DOPA is able to induce independent signaling routes apart from dopamine, for example in regulating cardiovascular activity (Goshima et al., 1986, 1988, 2014, 2019a; Kubo et al., 1992; Yue et al., 1994). Since L-DOPA is a potential ligand for GPR143, Goshima et al. proposed that L-DOPA itself, rather than dopamine, may function as a neurotransmitter *via* this receptor (Goshima et al., 1988, 2019a, 2019b; Nakamura et al., 1992). According to their studies, GPR143 colocalized with phosphorylated α-synuclein in Lewy bodies in the brains of PD patients (Goshima et al., 2019b). These Lewy bodies are abnormal protein aggregates and the presence of GPR143 in these inclusion bodies could alter activity of the receptor and dysregulate L-DOPA signaling. Alternatively, given the proposed role for GPR143 in organelle biogenesis in melanocytes, the presence of GPR143 may be due to its participation in endolysosomal protein trafficking.

Goshima et al. also observed GPR143-immunoreactive signals, in both control and PD brains, in the entire midbrain region including the substantia nigra pars compacta (Goshima et al., 2019b), a basal ganglia structure and contains dopaminergic neurons that produce neuromelanin (Rabey and Hefti, 1990). The loss of these dopaminergic neurons is characteristic of PD (Kim et al., 2003). Taken together, Goshima and co-workers suggest that GPR143 may be implicated in the pathogenesis of PD and could be targeted for development of therapeutics (Goshima et al., 2019a).

In a very recent publication Kasahara et al. present evidence that L-DOPA-induced neurogenesis in the hippocampus of mice that might be mediated by GPR143 (Kasahara et al., 2022). Lastly, GPR143 was also found to be upregulated together with other genes in autism spectrum disorder (Monfared et al., 2021).

### GPR143 and Macular Degeneration

Age-related macular degeneration (AMD), a multifactorial disease, leads to progressive vision loss (Lim et al., 2012; Mitchell et al., 2018). In early stages, RPE abnormalities are observed in the macular region of the retina followed by neovascular changes and finally total blindness (Coleman et al., 2008; Mitchell et al., 2018). Highly effective anti-neovascular agents such as vascular endothelial growth factor (VEGF)-inhibitors, applied by ocular injections, are used to treat AMD (Coleman et al., 2008; Lim et al., 2012; Bourne et al., 2013). Development of alternative treatments that require less invasive administration routes is therefore necessary (Figueroa and McKay, 2019).

An early study by Zhang et al. determined that Pigment Epithelium-Derived Factor (PEDF) can downregulate VEGF since decreased PEDF levels in the retina were shown to increase VEGF expression (Zhang et al., 2006). It was later discovered that there is extensive crosstalk between VEGF and PEDF that contributes to a neuroprotective effect (Falk et al., 2010).

PEDF was also found to be highly expressed in the RPE of young adults, with expression rapidly decreasing with onset of cell senescence (Tombran-Tink et al., 1995). In a study by Lopez et al., RPE cells were treated with high concentrations of L-DOPA and were shown to increase PEDF expression, which was attributed to GPR143 activity (Lopez et al., 2008). Furthermore, a retrospective study analyzing the medical history of AMD-patients found that those who were or had been on an L-DOPA regimen for movement disorders showed a later onset of AMD than those who had not received similar medication (Brilliant et al., 2016). If modulation of GPR143 could be employed to increase PEDF expression and thereby downregulate VEGF, the receptor has potential as a new target for the treatment of AMD (Falk et al., 2012).

### GPR143 and Cancer

In 2020, around 10 million deaths were accounted for from cancer worldwide and with an estimation of 11.4 million deaths in 2030, cancer is a leading cause of death (O’Callaghan, 2011; Ferlay et al., 2021). Several GPCRs have been linked to different cancers and their role in regulating tumorigenesis, proliferation, invasion and metastasis is slowly being defined. GPR143 has also been associated with multiple cancers including melanoma, non-melanoma skin cancer, breast cancer, uveal carcinoma, colorectal cancer (Bassi et al., 1995; Uhlén et al., 2015; Uhlen et al., 2017). In a recent bioinformatic analysis investigating unilateral and bilateral retinoblastoma microarrays, GPR143 was identified as a potential biomarker for the cancer (Zhao et al., 2021).

#### Melanoma and Skin Cancer

Malignant melanoma is one of the most aggressive cancers, with a highly metastatic potential and a predilection for developing resistance to therapies (Bai et al., 2014). Furthermore, in the US, melanoma is the sixth most common cancer type (Borden et al., 2021). Despite substantial efforts to develop targeted therapies, long-term success remains challenging (Li et al., 2011; Kozar et al., 2019; Alvarez-Artime et al., 2020; Berge et al., 2020; Cherepakhin et al., 2021).

GPR143 expression has been shown to correlate with prognosis in cutaneous melanoma and is a reliable biomarker for identifying immune infiltration (Xing et al., 2021). In addition, when Bai et al. used lentivirus constructs to express exogenous GPR143 in human melanoma cells, the amount of vector transfected was positively correlated with cell migration (Bai et al., 2014). This effect could be inhibited by siRNA-mediated GPR143 knockdown (Bai et al., 2014). GPR143 expression increased with progression towards metastasis (Fernandez et al., 2009; Bai et al., 2014). In addition, the study revealed that GPR143-induced melanoma cell metastasis activated the RAS/RAF/MEK/ERK signaling pathway (Bai et al., 2014). Bai *et al.* concluded from their findings, that GPR143 may serve as a metastasis-promoting gene in the progression of melanoma (Bai et al., 2014). Some melanosome-related proteins have been correlated with melanoma chemoresistance (Kottschade et al., 2016), for example the microphthalmia-associated transcription factor (MITF) was expressed at significantly higher levels in dacarbazine/temozolomide-resistant tumors when compared to sensitive tumors (Kottschade et al., 2016). Upregulation of GPR143 in chemoresistant melanoma may thus be due to upregulation of MITF, since MITF directly controls GPR143 expression (Vetrini et al., 2004; Kottschade et al., 2016).

#### Colorectal Cancer

Chromosome Xp22.2-22.3, where the *OA1* gene is localized, is a colorectal cancer risk locus (Spatz et al., 2004; Benoît et al., 2007; McBride, 2016). Colorectal cancer is the second leading cause of cancer death worldwide with 881 000 estimated deaths in 2018 (Bray et al., 2018; Dolin et al., 2021). This type of cancer is highly associated with age (Ellison et al., 2018; Siegel et al., 2020; Dolin et al., 2021). Aside from an unhealthy lifestyle, colorectal cancer can also develop due to genetic mutations (Munteanu and Mastalier, 2014). Genome-wide association studies also identified GPR143 and its neighbor SHROOM2 as susceptibility genes for cancer (Dunlop et al., 2012; Closa et al., 2014; Chen Z. et al., 2019). Closa et al. hypothesized that since GPR143 and SHROOM2 may both play a role in retinal pigmentation and patients with familial adenomatous polyposis syndrome can also develop benign RPE lesions (known as congenital hypertrophy of retinal pigment epithelium lesions) mutations in the region of Xp22.2 may increase the risk for developing colorectal cancer (Díaz-Llopis and Menezo, 1988; Galiatsatos and Foulkes, 2006; Closa et al., 2014, https://rarediseases.org/rare-diseases/familial-adenomatous-polyposis/, accessed 4 January 2022).

### GPR143 and Nicotine-Induced Behavior

Nicotine administration increases the release of L-DOPA in the nucleus accumbens of rats and promotes locomotion (walking, chewing etc.). The effects of nicotine can be suppressed by an L-DOPA antagonist, thus endogenous L-DOPA may play a role in nicotine-induced behavior (Goshima et al., 1996; Izawa et al., 2006). Since L-DOPA is a potential ligand for GPR143, Masukawa *et al.* investigated potential genetic association between GPR143 polymorphisms and smoking behavior in Japanese individuals (Masukawa et al., 2020). Their results pointed to rs6640499, a single-nucleotide polymorphism of GPR143, which was associated with traits of smoking behaviors such as number of cigarettes smoked per day (Masukawa et al., 2020). Surprisingly, in this study, Masukawa et al. found that nicotine reduced locomotion in WT mice (contrary to previous findings). GPR143 KO mice did not show a similar reduction. When investigating nicotine-induced reward related behavior, a significant difference was noted between WT and GPR143 KO mice at low nicotine doses, with reward behavior attenuated in the mutant mice (Masukawa et al., 2020).

## GPR143 Interaction Partners

### Melanosomal Proteins

The many hypotheses regarding GPR143 and its precise function may be due to its promiscuity in forming protein-protein-interactions (PPI) with different partners. GPR143 was initially shown to interact with melanocyte proteins, potentially while GPR143 is trafficked to melanosomes through endolysosomal/melanosomal pathways similar to other melanosomal proteins. (Winder et al., 1994). For instance the melanocyte protein melanoma antigen recognized by T-cells (MART-1), also known as melan-A (Kawakami et al., 1994), was shown to interact with GPR143 (Giordano et al., 2009). Giordano *et al.* showed, that inactivation of MART-1 would lead to a decreased stability of GPR143 and other defects in premelanosome biogenesis, suggesting that MART-1 acts as an escort for GPR143 at early stages of melanosome formation (Giordano et al., 2009).

De Filippo et al*.* GPR143 found that GPR143 interacts with tyrosinase, the rate-limiting enzyme of melanin synthesis (De Filippo et al., 2017b). The interaction may be required to effectively control melanosome maturation. Earlier *in vivo* studies had shown that KO of both GPR143 and tyrosinase would prevent macromelanosomes formation (Cortese et al., 2005).

GPR143 was also shown to bind tubulin in a study by Palmisano et al., where GPR143 knockout mice showed abnormal melanosome distribution in RPE and melanocytes (Palmisano et al., 2008). This defect was rescued when GPR143 was re-expressed following transfection suggesting that melanosomes require GPR143 for microtubule-mediated distribution from the perinuclear area to the cell periphery (Palmisano et al., 2008). Since tyrosinase would be expected to bind GPR143 in the lumen of the melanosome and tubulin in the intercellular space, GPR143 may interact with proteins/ligands from both membrane faces (Schiaffino and Tacchetti, 2005).

In GPR143 deficient cells, fewer but larger marcomelanosomes are observed (Sitaram and Marks, 2012). As the precise regulation of melanosome size is not fully understood, the role of master regulator of melanocyte differentiation, MITF was investigated in association with GPR143 (Falletta et al., 2014). It was reported that GPR143 is able to interact with MITF through a feedback loop, being both a regulator and target. GRP143 may also impact other genes regulated by MITF (Vetrini et al., 2004; Falletta et al., 2014). For example, the melanosome scaffold protein (PMEL) required for melanosome maturation, was functionally linked to GPR143. Loss of GPR143 function reduced both basal expression of MITF and α-melanocyte-stimulating hormone-dependent induction of MITF. Hence, expression of PMEL was also reduced (Falletta et al., 2014). The authors concluded that GPR143 modulates melanosome maturation by enhancing MITF expression and coordinating melansomes size and number in a quality control-manner (Falletta et al., 2014).

### G Protein-Coupled Receptors

Masukawa et al. determined that α_1b_-adrenoceptor and GPR143 are able to form functional heteromers that modulate noradrenaline-mediated regulation of blood pressure through L-DOPA (Masukawa et al., 2017). In their study, mice were given an infusion of phenylephrine (an α_1b_-adrenoceptor agonist) that caused a transient increase in blood pressure in wildtype but not GPR143-mutant mice (Masukawa et al., 2017; Goshima et al., 2019a). Interaction was further confirmed by colocalization in FRET and *in situ* proximity ligation assays, furthermore, the binding affinity between the two receptors was enhanced in immunoprecipitation assays following pretreatment with L-DOPA (Masukawa et al., 2017) (see chapter 4.2. above).

We demonstrated that GPR143 forms functional dimers with D_2_R and D_3_R, which results in significant reduction of D_2/3_R response towards dopamine. GRP143 may exert its effect either by changing DR affinity for dopamine or by delaying the delivery of the DRs to the plasma membrane (Bueschbell et al., 2021). The link between the pigmentary system and dopaminergic signaling may be critical for optic tract development, since GPR143 function and tyrosinase activity could facilitate formation of an L-DOPA concentration gradient that allows for correct nerve projection during development (Chen et al., 2015; Chagraoui et al., 2019; Bueschbell et al., 2021).

GPR143 and its effect on DRs may play a role in the pathology of AMD due to their effect on dopamine in retinal neurons and RPE where L-DOPA is converted to dopamine (Figueroa and McKay, 2019).

## Conclusion and Future Aspects

GPR143, first discovered as the product of the gene mutated in ocular albinism 1, is an enigmatic receptor that is unusual in many ways. It is expressed intracellularly and cannot be readily classified into a particular GPCR family. Neither the signaling pathways nor the exact functions of the protein have been defined, particularly with regard to its role in disease pathophysiology. There are limited pharmacological tools available to analyze GPR143 pharmacological and biological functions. The aim of our review therefore was to shine light on this unique receptor by collecting relevant information about GPR143, focusing not only on a specific function, but rather aiming to bring together all facets of the potentially diverse roles the protein may play. We highlight structural features which suggest GPR143 is a class A GPCR since it shares only few features with class B receptors, as well as the involvement of the receptor in various diseases. GPR143 is on one hand important for early development of the eye, causing ocular albinism type 1, if mutated, and on the other hand implicated in a variety of degenerative diseases, such as macular degeneration and Parkinsons disease and potentially even plays a role in susceptibility and progression of cancers. GPR143 is not a mainstream receptor in terms of drug development. Nevertheless, it is a prime example of an orphan receptor with therapeutic potential. Much more research is needed to fully understand the (patho)physiology of GPR143.
