# Supplementary material for: The Many Faces of G Protein-Coupled Receptor 143, an Atypical Intracellular Receptor
Source: Front Mol Biosci. 2022 Apr 12;9:873777. doi: 10.3389/fmolb.2022.873777 (PMC9039016; doi:10.3389/fmolb.2022.873777)
Supplement: Supplementary file 7 [file DataSheet1.DOCX]

Supplementary Material

# References for Tables S1-6 for G protein-coupled receptor 143 mutations

[Addio, M. d’, A. Pizzigoni, M. T. Bassi, C. Baschirotto, C. Valetti, B. Incerti, M. Clementi, M. De Luca, A. Ballabio, and M. V. Schiaffino. 2000. “Defective Intracellular Transport and Processing of OA1 Is a Major Cause of Ocular Albinism Type 1.” *Human Molecular Genetics* 9 (20): 3011–18. https://doi.org/](http://paperpile.com/b/pec3nZ/XcEa)[10.1093/hmg/9.20.3011](http://dx.doi.org/10.1093/hmg/9.20.3011)[.](http://paperpile.com/b/pec3nZ/XcEa)

[Bassi, M. T., A. A. Bergen, P. Bitoun, S. J. Charles, M. Clementi, R. Gosselin, J. Hurst, et al. 2001. “Diverse Prevalence of Large Deletions within the OA1 Gene in Ocular Albinism Type 1 Patients from Europe and North America.” *Human Genetics* 108 (1): 51–54. https://doi.org/](http://paperpile.com/b/pec3nZ/PVtQ)[10.1007/s004390000440](http://dx.doi.org/10.1007/s004390000440)[.](http://paperpile.com/b/pec3nZ/PVtQ)

[Bassi, M. T., R. S. Ramesar, B. Caciotti, I. M. Winship, A. De Grandi, M. Riboni, P. L. Townes, P. Beighton, A. Ballabio, and G. Borsani. 1999. “X-Linked Late-Onset Sensorineural Deafness Caused by a Deletion Involving OA1 and a Novel Gene Containing WD-40 Repeats.” *American Journal of Human Genetics* 64 (6): 1604–16. https://doi.org/](http://paperpile.com/b/pec3nZ/3UJv)[10.1086/302408](http://dx.doi.org/10.1086/302408)[.](http://paperpile.com/b/pec3nZ/3UJv)

[Bassi, M. T., M. V. Schiaffino, A. Renieri, F. De Nigris, L. Galli, M. Bruttini, M. Gebbia, A. A. Bergen, R. A. Lewis, and A. Ballabio. 1995. “Cloning of the Gene for Ocular Albinism Type 1 from the Distal Short Arm of the X Chromosome.” *Nature Genetics* 10 (1): 13–19. https://doi.org/](http://paperpile.com/b/pec3nZ/KWYT)[10.1038/ng0595-13](http://dx.doi.org/10.1038/ng0595-13)[.](http://paperpile.com/b/pec3nZ/KWYT)

[Burns, W. N., M. V. Schiaffino, and R. A. Lewis. 1998. “Repeated Transmission of X-Linked Ocular Albinism Type 1 by a Carrier Oocyte Donor.” *Fertility and Sterility* 70 (6): 1169–72. https://doi.org/](http://paperpile.com/b/pec3nZ/1YeW)[10.1016/s0015-0282(98)00387-2](http://dx.doi.org/10.1016/s0015-0282(98)00387-2)[.](http://paperpile.com/b/pec3nZ/1YeW)

[Cai, C. Y., H. Zhu, W. Shi, L. Su, O. Shi, C. Q. Cai, C. Ling, and W. D. Li. 2013. “A Novel Splicing Site Mutation of the GPR143 Gene in a Chinese X-Linked Ocular Albinism Pedigree.” *Genetics and Molecular Research: GMR* 12 (4): 5673–79. https://doi.org/](http://paperpile.com/b/pec3nZ/sxNc)[10.4238/2013.November.18.16](http://dx.doi.org/10.4238/2013.November.18.16)[.](http://paperpile.com/b/pec3nZ/sxNc)

[Camand, Olivier, Sandrine Boutboul, Laurence Arbogast, Olivier Roche, Claude Sternberg, Joanne Sutherland, Alex Levin, et al. 2003. “Mutational Analysis of the OA1 Gene in Ocular Albinism.” *Ophthalmic Genetics* 24 (3): 167–73. https://doi.org/](http://paperpile.com/b/pec3nZ/VJXh)[10.1076/opge.24.3.167.15605](http://dx.doi.org/10.1076/opge.24.3.167.15605)[.](http://paperpile.com/b/pec3nZ/VJXh)

[Campbell, Patrick, Jamie M. Ellingford, Neil R. A. Parry, Tracy Fletcher, Simon C. Ramsden, Theodora Gale, Georgina Hall, et al. 2019. “Clinical and Genetic Variability in Children with Partial Albinism.” *Scientific Reports* 9 (1): 16576. https://doi.org/](http://paperpile.com/b/pec3nZ/sFeN)[10.1038/s41598-019-51768-8](http://dx.doi.org/10.1038/s41598-019-51768-8)[.](http://paperpile.com/b/pec3nZ/sFeN)

[Carss, Keren J., Gavin Arno, Marie Erwood, Jonathan Stephens, Alba Sanchis-Juan, Sarah Hull, Karyn Megy, et al. 2017. “Comprehensive Rare Variant Analysis via Whole-Genome Sequencing to Determine the Molecular Pathology of Inherited Retinal Disease.” *American Journal of Human Genetics* 100 (1): 75–90. https://doi.org/](http://paperpile.com/b/pec3nZ/F7PX)[10.1016/j.ajhg.2016.12.003](http://dx.doi.org/10.1016/j.ajhg.2016.12.003)[.](http://paperpile.com/b/pec3nZ/F7PX)

[Chan, Hwei Wuen, Elena R. Schiff, Vijay K. Tailor, Samantha Malka, Magella M. Neveu, Maria Theodorou, and Mariya Moosajee. 2021. “Prospective Study of the Phenotypic and Mutational Spectrum of Ocular Albinism and Oculocutaneous Albinism.” *Genes* 12 (4). https://doi.org/](http://paperpile.com/b/pec3nZ/BIld)[10.3390/genes12040508](http://dx.doi.org/10.3390/genes12040508)[.](http://paperpile.com/b/pec3nZ/BIld)

[“COGR.” n.d. Accessed February 9, 2022.](http://paperpile.com/b/pec3nZ/HJmI) <http://opengenetics.ca/>[.](http://paperpile.com/b/pec3nZ/HJmI)

[Fang, Shaohua, Xiangming Guo, Xiaoyun Jia, Xueshan Xiao, Shiqiang Li, and Qingjiong Zhang. 2008. “Novel GPR143 Mutations and Clinical Characteristics in Six Chinese Families with X-Linked Ocular Albinism.” *Molecular Vision* 14 (October): 1974–82.](http://paperpile.com/b/pec3nZ/Jv5C) <https://www.ncbi.nlm.nih.gov/pubmed/18978956>[.](http://paperpile.com/b/pec3nZ/Jv5C)

[Gao, Xuhui, Tiecheng Liu, Xuan Cheng, Aiai Dai, Wei Liu, Runpu Li, and Maonian Zhang. 2020. “A Novel GPR143 Mutation in a Chinese Family with X‑linked Ocular Albinism Type 1.” *Molecular Medicine Reports* 21 (1): 240–48. https://doi.org/](http://paperpile.com/b/pec3nZ/ZzB1)[10.3892/mmr.2019.10813](http://dx.doi.org/10.3892/mmr.2019.10813)[.](http://paperpile.com/b/pec3nZ/ZzB1)

[Han, Ruifang, Xiaojuan Wang, Dongjie Wang, Liming Wang, Zhongfang Yuan, Ming Ying, and Ningdong Li. 2015. “GPR143 Gene Mutations in Five Chinese Families with X-Linked Congenital Nystagmus.” *Scientific Reports* 5 (July): 12031. https://doi.org/](http://paperpile.com/b/pec3nZ/bKbM)[10.1038/srep12031](http://dx.doi.org/10.1038/srep12031)[.](http://paperpile.com/b/pec3nZ/bKbM)

[Hegde, M., R. A. Lewis, and C. S. Richards. 2002. “Diagnostic DNA Testing for X-Linked Ocular Albinism (OA1) with a Hierarchical Mutation Screening Protocol.” *Genetic Testing* 6 (1): 7–14. https://doi.org/](http://paperpile.com/b/pec3nZ/bzHP)[10.1089/109065702760093852](http://dx.doi.org/10.1089/109065702760093852)[.](http://paperpile.com/b/pec3nZ/bzHP)

[Hu, Junjie, Desheng Liang, Jinjie Xue, Jing Liu, and Lingqian Wu. 2011. “A Novel GPR143 Splicing Mutation in a Chinese Family with X-Linked Congenital Nystagmus.” *Molecular Vision* 17 (March): 715–22.](http://paperpile.com/b/pec3nZ/6RDC) <https://www.ncbi.nlm.nih.gov/pubmed/21423867>[.](http://paperpile.com/b/pec3nZ/6RDC)

[Iannaccone, Alessandro, Kevin T. Gallaher, Janda Buchholz, Barbara J. Jennings, Maureen Neitz, and D. J. Sidjanin. 2007. “Identification of Two Novel Mutations in Families with X-Linked Ocular Albinism.” *Molecular Vision* 13 (October): 1856–61.](http://paperpile.com/b/pec3nZ/7eiX) <https://www.ncbi.nlm.nih.gov/pubmed/17960122>[.](http://paperpile.com/b/pec3nZ/7eiX)

[Iarossi, Giancarlo, Andrea Maria Coppè, Chiara Passarelli, Paolo Enrico Maltese, Lorenzo Sinibaldi, Alessandro Cappelli, Sarah Cetola, Antonio Novelli, and Luca Buzzonetti. 2021. “Blue Cone Monochromatism with Foveal Hypoplasia Caused by the Concomitant Effect of Variants in OPN1LW/OPN1MW and GPR143 Genes.” *International Journal of Molecular Sciences* 22 (16). https://doi.org/](http://paperpile.com/b/pec3nZ/Xa0L)[10.3390/ijms22168617](http://dx.doi.org/10.3390/ijms22168617)[.](http://paperpile.com/b/pec3nZ/Xa0L)

[Jiang, Jingjing, Likun Yang, Hui Li, Lijuan Huang, and Ningdong Li. 2019. “Evaluation of the Iris Thickness Changes for the Chinese Families with GPR143 Gene Mutations.” *Experimental Eye Research* 189 (107819): 107819. https://doi.org/](http://paperpile.com/b/pec3nZ/Sd6F)[10.1016/j.exer.2019.107819](http://dx.doi.org/10.1016/j.exer.2019.107819)[.](http://paperpile.com/b/pec3nZ/Sd6F)

[Jia, Xiuhua, Jin Yuan, Xiaoyun Jia, Shiqi Ling, Shiqiang Li, and Xiangming Guo. 2017. “GPR143 Mutations in Chinese Patients with Ocular Albinism Type 1.” *Molecular Medicine Reports* 15 (5): 3069–75. https://doi.org/](http://paperpile.com/b/pec3nZ/rGqS)[10.3892/mmr.2017.6366](http://dx.doi.org/10.3892/mmr.2017.6366)[.](http://paperpile.com/b/pec3nZ/rGqS)

[Jie, Li, Xing Yasi, Li Zhanrong, Lu Xiaonan, and Dai Shuzhen. n.d. “Pathogenic Gene Screening and Phenotypic Analysis of Six Albinism Families.” Accessed February 9, 2022. https://doi.org/](http://paperpile.com/b/pec3nZ/vQ9a)[10.3760/cma.j.issn.1005-1015.2018.06.003](http://dx.doi.org/10.3760/cma.j.issn.1005-1015.2018.06.003)[.](http://paperpile.com/b/pec3nZ/vQ9a)

[Jung, Jae-Ho, Eun Hye Oh, Jin-Hong Shin, Hyang-Sook Kim, Seo Young Choi, Kwang-Dong Choi, Changwook Lee, and Jae-Hwan Choi. 2018. “Identification of a Novel GPR143 Mutation in X-Linked Ocular Albinism with Marked Intrafamilial Phenotypic Variability.” *Journal of Genetics* 97 (5): 1479–84. https://doi.org/](http://paperpile.com/b/pec3nZ/q2U7)[10.1007/s12041-018-1024-7](http://dx.doi.org/10.1007/s12041-018-1024-7)[.](http://paperpile.com/b/pec3nZ/q2U7)

[Khan, A. O., M. Tamimi, S. Lenzner, and H. J. Bolz. 2016. “Hermansky-Pudlak Syndrome Genes Are Frequently Mutated in Patients with Albinism from the Arabian Peninsula.” *Clinical Genetics* 90 (1): 96–98. https://doi.org/](http://paperpile.com/b/pec3nZ/imRz)[10.1111/cge.12715](http://dx.doi.org/10.1111/cge.12715)[.](http://paperpile.com/b/pec3nZ/imRz)

[Khan, Kamron N., Emma C. Lord, Gavin Arno, Farrah Islam, Keren J. Carss, Flucy Raymond, Carmel Toomes, et al. 2018. “DETAILED RETINAL IMAGING IN CARRIERS OF OCULAR ALBINISM.” *Retina*  38 (3): 620–28. https://doi.org/](http://paperpile.com/b/pec3nZ/jbHp)[10.1097/IAE.0000000000001570](http://dx.doi.org/10.1097/IAE.0000000000001570)[.](http://paperpile.com/b/pec3nZ/jbHp)

[Lam, B. L., J. H. Fingert, B. C. Shutt, E. M. Singleton, L. M. Merin, H. H. Brown, V. C. Sheffield, and E. M. Stone. 1997. “Clinical and Molecular Characterization of a Family Affected with X-Linked Ocular Albinism (OA1).” *Ophthalmic Genetics* 18 (4): 175–84. https://doi.org/](http://paperpile.com/b/pec3nZ/S5eE)[10.3109/13816819709041432](http://dx.doi.org/10.3109/13816819709041432)[.](http://paperpile.com/b/pec3nZ/S5eE)

[Lasseaux, Eulalie, Claudio Plaisant, Vincent Michaud, Perrine Pennamen, Aurelien Trimouille, Laetitia Gaston, Solène Monfermé, et al. 2018. “Molecular Characterization of a Series of 990 Index Patients with Albinism.” *Pigment Cell & Melanoma Research* 31 (4): 466–74. https://doi.org/](http://paperpile.com/b/pec3nZ/H3e6)[10.1111/pcmr.12688](http://dx.doi.org/10.1111/pcmr.12688)[.](http://paperpile.com/b/pec3nZ/H3e6)

[Lauronen, L., R. Jalkanen, J. Huttunen, E. Carlsson, S. Tuupanen, S. Lindh, H. Forsius, E-M Sankila, and T. Alitalo. 2005. “Abnormal Crossing of the Optic Fibres Shown by Evoked Magnetic Fields in Patients with Ocular Albinism with a Novel Mutation in the OA1 Gene.” *The British Journal of Ophthalmology* 89 (7): 820–24. https://doi.org/](http://paperpile.com/b/pec3nZ/kF7F)[10.1136/bjo.2004.060582](http://dx.doi.org/10.1136/bjo.2004.060582)[.](http://paperpile.com/b/pec3nZ/kF7F)

[Lee, Winston, Kaspar Schuerch, Yajing Xie, Jana Zernant, Stephen H. Tsang, Janet R. Sparrow, and Rando Allikmets. 2016. “Simultaneous Expression of ABCA4 and GPR143 Mutations: A Complex Phenotypic Manifestation.” *Investigative Ophthalmology & Visual Science* 57 (7): 3409–15. https://doi.org/](http://paperpile.com/b/pec3nZ/wOQW)[10.1167/iovs.16-19621](http://dx.doi.org/10.1167/iovs.16-19621)[.](http://paperpile.com/b/pec3nZ/wOQW)

[Lek, Monkol, Konrad J. Karczewski, Eric V. Minikel, Kaitlin E. Samocha, Eric Banks, Timothy Fennell, Anne H. O’Donnell-Luria, et al. 2016. “Analysis of Protein-Coding Genetic Variation in 60,706 Humans.” *Nature* 536 (7616): 285–91. https://doi.org/](http://paperpile.com/b/pec3nZ/MtOH)[10.1038/nature19057](http://dx.doi.org/10.1038/nature19057)[.](http://paperpile.com/b/pec3nZ/MtOH)

[Li, Ningdong, and Jingjing Jiang. 2018. “Analysis of GPR143 Gene Mutations in Five Chinese Families with the Ocular Albinism Type I.” *Investigative Ophthalmology & Visual Science* 59 (9): 1030–1030.](http://paperpile.com/b/pec3nZ/gKaA) <https://iovs.arvojournals.org/article.aspx?articleid=2689519>[.](http://paperpile.com/b/pec3nZ/gKaA)

[Liu, Jing, Yanlei Jia, Lejin Wang, and Juan Bu. 2016. “A Previously Unidentified Deletion in G Protein-Coupled Receptor 143 Causing X-Linked Congenital Nystagmus in a Chinese Family.” *Indian Journal of Ophthalmology* 64 (11): 813–17. https://doi.org/](http://paperpile.com/b/pec3nZ/h6AT)[10.4103/0301-4738.195593](http://dx.doi.org/10.4103/0301-4738.195593)[.](http://paperpile.com/b/pec3nZ/h6AT)

[Liu, Jing Yu, Xiang Ren, Xiufeng Yang, Tangying Guo, Qi Yao, Lin Li, Xiaohua Dai, et al. 2007. “Identification of a Novel GPR143 Mutation in a Large Chinese Family with Congenital Nystagmus as the Most Prominent and Consistent Manifestation.” *Journal of Human Genetics* 52 (6): 565–70. https://doi.org/](http://paperpile.com/b/pec3nZ/eaLo)[10.1007/s10038-007-0152-3](http://dx.doi.org/10.1007/s10038-007-0152-3)[.](http://paperpile.com/b/pec3nZ/eaLo)

[“Locus Variants.” n.d. Accessed February 9, 2022.](http://paperpile.com/b/pec3nZ/o2Zq) [http://phencode.bx.psu.edu/cgi-bin/phencode/phencode?build=hg19&id=RISN_OA1:c.730A>G](http://phencode.bx.psu.edu/cgi-bin/phencode/phencode?build=hg19&id=RISN_OA1:c.730A%3EG)[.](http://paperpile.com/b/pec3nZ/o2Zq)

[Mao, Xiying, Mingkang Chen, Yan Yu, Qinghuai Liu, Songtao Yuan, and Wen Fan. 2021. “Identification of a Novel GPR143 Mutation in a Large Chinese Family with Isolated Foveal Hypoplasia.” *BMC Ophthalmology* 21 (1): 156. https://doi.org/](http://paperpile.com/b/pec3nZ/ak3n)[10.1186/s12886-021-01905-7](http://dx.doi.org/10.1186/s12886-021-01905-7)[.](http://paperpile.com/b/pec3nZ/ak3n)

[Marti, Aurélie, Eulalie Lasseaux, Khaled Ezzedine, Christine Léauté-Labrèze, Franck Boralevi, Clément Paya, Valentine Coste, et al. 2018. “Lessons of a Day Hospital: Comprehensive Assessment of Patients with Albinism in a European Setting.” *Pigment Cell & Melanoma Research* 31 (2): 318–29. https://doi.org/](http://paperpile.com/b/pec3nZ/0dQs)[10.1111/pcmr.12651](http://dx.doi.org/10.1111/pcmr.12651)[.](http://paperpile.com/b/pec3nZ/0dQs)

[Martinez-Garcia, Monica, M. J. Trujillo-Tiebas, C. Villaverde, M. A. López-Martínez, and C. Ayuso. 2009. “Novel Human Pathological Mutations. Gene Symbol: OA1. Disease: Albinism, Ocular.” *Human Genetics* 125 (3): 349.](http://paperpile.com/b/pec3nZ/JgVZ) <https://www.ncbi.nlm.nih.gov/pubmed/19320034>[.](http://paperpile.com/b/pec3nZ/JgVZ)

[Mauri, Lucia, Emanuela Manfredini, Alessandra Del Longo, Emanuela Veniani, Manuela Scarcello, Roberta Terrana, Adriano Egidio Radaelli, et al. 2017. “Clinical Evaluation and Molecular Screening of a Large Consecutive Series of Albino Patients.” *Journal of Human Genetics* 62 (2): 277–90. https://doi.org/](http://paperpile.com/b/pec3nZ/Ph76)[10.1038/jhg.2016.123](http://dx.doi.org/10.1038/jhg.2016.123)[.](http://paperpile.com/b/pec3nZ/Ph76)

[Mayeur, Hélène, Olivier Roche, Christelle Vêtu, Carolina Jaliffa, Dominique Marchant, Hélène Dollfus, Dominique Bonneau, et al. 2006. “Eight Previously Unidentified Mutations Found in the OA1 Ocular Albinism Gene.” *BMC Medical Genetics* 7 (1): 41. https://doi.org/](http://paperpile.com/b/pec3nZ/FImX)[10.1186/1471-2350-7-41](http://dx.doi.org/10.1186/1471-2350-7-41)[.](http://paperpile.com/b/pec3nZ/FImX)

[Micale, Lucia, Bartolomeo Augello, Carmela Fusco, Maria Giuseppina Turturo, Matteo Granatiero, Maria Rosaria Piemontese, Leopoldo Zelante, Antonella Cecconi, and Giuseppe Merla. 2009. “GPR143 Mutational Analysis in Two Italian Families with X-Linked Ocular Albinism.” *Genetic Testing and Molecular Biomarkers* 13 (4): 527–31. https://doi.org/](http://paperpile.com/b/pec3nZ/9AJa)[10.1089/gtmb.2009.0030](http://dx.doi.org/10.1089/gtmb.2009.0030)[.](http://paperpile.com/b/pec3nZ/9AJa)

[Montoya Delgado, M. J., M. C. Astiazarán, F. Casanova Imken, A. Ramírez Estudillo, and Á. Hernández Vázquez. 2019. “Ocular Albinism with Mutation in GPR143: Findings in Wide-Field Autofluorescence and Optical Coherence Tomography.” *Archivos de La Sociedad Española de Oftalmología (English Edition)* 94 (6): 288–92. https://doi.org/](http://paperpile.com/b/pec3nZ/8IEY)[10.1016/j.oftale.2019.01.012](http://dx.doi.org/10.1016/j.oftale.2019.01.012)[.](http://paperpile.com/b/pec3nZ/8IEY)

[Morice-Picard, Fanny, Eulalie Lasseaux, Claudio Plaisant, Dorothée Cailley, Julie Bouron, Caroline Rooryck, Didier Lacombe, et al. 2016. “Albinism in a Patient with Mutations at Both the OA1 and OCA3 Loci.” *Pigment Cell & Melanoma Research* 29 (1): 107–9. https://doi.org/](http://paperpile.com/b/pec3nZ/hwJ2)[10.1111/pcmr.12408](http://dx.doi.org/10.1111/pcmr.12408)[.](http://paperpile.com/b/pec3nZ/hwJ2)

[Naruto, Takuya, Nobuhiko Okamoto, Kiyoshi Masuda, Takao Endo, Yoshikazu Hatsukawa, Tomohiro Kohmoto, and Issei Imoto. 2015. “Deep Intronic GPR143 Mutation in a Japanese Family with Ocular Albinism.” *Scientific Reports* 5 (June): 11334. https://doi.org/](http://paperpile.com/b/pec3nZ/BMdu)[10.1038/srep11334](http://dx.doi.org/10.1038/srep11334)[.](http://paperpile.com/b/pec3nZ/BMdu)

[Nykamp, Keith, Michael Anderson, Martin Powers, John Garcia, Blanca Herrera, Yuan-Yuan Ho, Yuya Kobayashi, et al. 2017. “Sherloc: A Comprehensive Refinement of the ACMG-AMP Variant Classification Criteria.” *Genetics in Medicine: Official Journal of the American College of Medical Genetics* 19 (10): 1105–17. https://doi.org/](http://paperpile.com/b/pec3nZ/kfel)[10.1038/gim.2017.37](http://dx.doi.org/10.1038/gim.2017.37)[.](http://paperpile.com/b/pec3nZ/kfel)

[Oetting, William S. 2002. “New Insights into Ocular Albinism Type 1 (OA1): Mutations and Polymorphisms of the OA1 Gene.” *Human Mutation* 19 (2): 85–92. https://doi.org/](http://paperpile.com/b/pec3nZ/msNd)[10.1002/humu.10034](http://dx.doi.org/10.1002/humu.10034)[.](http://paperpile.com/b/pec3nZ/msNd)

[Paavo, Maarjaliis, Jin Zhao, Hye Jin Kim, Winston Lee, Jana Zernant, Carolyn Cai, Rando Allikmets, Stephen H. Tsang, and Janet R. Sparrow. 2018. “Mutations in GPR143/OA1 and ABCA4 Inform Interpretations of Short-Wavelength and Near-Infrared Fundus Autofluorescence.” *Investigative Ophthalmology & Visual Science* 59 (6): 2459–69. https://doi.org/](http://paperpile.com/b/pec3nZ/GsFG)[10.1167/iovs.18-24213](http://dx.doi.org/10.1167/iovs.18-24213)[.](http://paperpile.com/b/pec3nZ/GsFG)

[Pan, Qihao, Changxian Yi, Tingting Xu, Jinsong Liu, Xiangyi Jing, Bin Hu, and Yiming Wang. 2016. “A Novel Mutation, c.494C>A (p.Ala165Asp), in the GPR143 Gene Causes a Mild Phenotype in a Chinese X-Linked Ocular Albinism Patient.” *Acta Ophthalmologica* 94 (4): 417–18. https://doi.org/](http://paperpile.com/b/pec3nZ/mW4J)[10.1111/aos.12854](http://dx.doi.org/10.1111/aos.12854)[.](http://paperpile.com/b/pec3nZ/mW4J)

[Peng, Yuanyuan, Yan Meng, Zheng Wang, Mei Qin, Xiaoqiao Li, Yan Dian, and Shangzhi Huang. 2009. “A Novel GPR143 Duplication Mutation in a Chinese Family with X-Linked Congenital Nystagmus.” *Molecular Vision* 15 (April): 810–14.](http://paperpile.com/b/pec3nZ/RnXN) <https://www.ncbi.nlm.nih.gov/pubmed/19390656>[.](http://paperpile.com/b/pec3nZ/RnXN)

[Preising, Markus N., Hedwig Forster, Miriam Gonser, and Birgit Lorenz. 2011. “Screening of TYR, OCA2, GPR143, and MC1R in Patients with Congenital Nystagmus, Macular Hypoplasia, and Fundus Hypopigmentation Indicating Albinism.” *Molecular Vision* 17 (April): 939–48.](http://paperpile.com/b/pec3nZ/JGUA) <https://www.ncbi.nlm.nih.gov/pubmed/21541274>[.](http://paperpile.com/b/pec3nZ/JGUA)

[Rim, John Hoon, Seung-Tae Lee, Heon Yung Gee, Byung Joo Lee, Jong Rak Choi, Hye Won Park, Sueng-Han Han, and Jinu Han. 2017. “Accuracy of Next-Generation Sequencing for Molecular Diagnosis in Patients With Infantile Nystagmus Syndrome.” *JAMA Ophthalmology* 135 (12): 1376–85. https://doi.org/](http://paperpile.com/b/pec3nZ/6Iw2)[10.1001/jamaophthalmol.2017.4859](http://dx.doi.org/10.1001/jamaophthalmol.2017.4859)[.](http://paperpile.com/b/pec3nZ/6Iw2)

[Roma, Cristin, Paola Ferrante, Ombretta Guardiola, Andrea Ballabio, and Massimo Zollo. 2007. “New Mutations Identified in the Ocular Albinism Type 1 Gene.” *Gene* 402 (1-2): 20–27. https://doi.org/](http://paperpile.com/b/pec3nZ/xmE6)[10.1016/j.gene.2007.07.020](http://dx.doi.org/10.1016/j.gene.2007.07.020)[.](http://paperpile.com/b/pec3nZ/xmE6)

[Rosenberg, T., and M. Schwartz. 1998. “X-Linked Ocular Albinism: Prevalence and Mutations--a National Study.” *European Journal of Human Genetics: EJHG* 6 (6): 570–77. https://doi.org/](http://paperpile.com/b/pec3nZ/4LAB)[10.1038/sj.ejhg.5200226](http://dx.doi.org/10.1038/sj.ejhg.5200226)[.](http://paperpile.com/b/pec3nZ/4LAB)

[Rudolph, Günther, Alfons Meindl, Martin Bechmann, Hermann-Dieter Schworm, Helene Achatz, Klaus-Peter Boergen, Anselm Kampik, Thomas Berninger, and Thomas Meitinger. 2001. “X-Linked Ocular Albinism (Nettleship-Falls): A Novel 29-Bp Deletion in Exon 1.” *Graefe’s Archive for Clinical and Experimental Ophthalmology = Albrecht von Graefes Archiv Fur Klinische Und Experimentelle Ophthalmologie* 239 (3): 167–72. https://doi.org/](http://paperpile.com/b/pec3nZ/CrUI)[10.1007/s004170000234](http://dx.doi.org/10.1007/s004170000234)[.](http://paperpile.com/b/pec3nZ/CrUI)

[Schaefer, L., G. B. Ferrero, A. Grillo, M. T. Bassi, E. J. Roth, M. C. Wapenaar, G. J. van Ommen, et al. 1993. “A High Resolution Deletion Map of Human Chromosome Xp22.” *Nature Genetics* 4 (3): 272–79. https://doi.org/](http://paperpile.com/b/pec3nZ/VlrY)[10.1038/ng0793-272](http://dx.doi.org/10.1038/ng0793-272)[.](http://paperpile.com/b/pec3nZ/VlrY)

[Schiaffino, M. V., M. T. Bassi, L. Galli, A. Renieri, M. Bruttini, F. De Nigris, A. A. Bergen, S. J. Charles, J. R. Yates, and A. Meindl. 1995. “Analysis of the OA1 Gene Reveals Mutations in Only One-Third of Patients with X-Linked Ocular Albinism.” *Human Molecular Genetics* 4 (12): 2319–25. https://doi.org/](http://paperpile.com/b/pec3nZ/bAk6)[10.1093/hmg/4.12.2319](http://dx.doi.org/10.1093/hmg/4.12.2319)[.](http://paperpile.com/b/pec3nZ/bAk6)

[Schnur, R. E., M. Gao, P. A. Wick, M. Keller, P. J. Benke, M. J. Edwards, A. W. Grix, et al. 1998. “OA1 Mutations and Deletions in X-Linked Ocular Albinism.” *American Journal of Human Genetics* 62 (4): 800–809. https://doi.org/](http://paperpile.com/b/pec3nZ/DOuQ)[10.1086/301776](http://dx.doi.org/10.1086/301776)[.](http://paperpile.com/b/pec3nZ/DOuQ)

[Schnur, R. E., P. A. Wick, C. Bailey, T. Rebbeck, R. G. Weleber, J. Wagstaff, A. W. Grix, R. A. Pagon, A. Hockey, and M. J. Edwards. 1994. “Phenotypic Variability in X-Linked Ocular Albinism: Relationship to Linkage Genotypes.” *American Journal of Human Genetics* 55 (3): 484–96.](http://paperpile.com/b/pec3nZ/R8d9) <https://www.ncbi.nlm.nih.gov/pubmed/7915878>[.](http://paperpile.com/b/pec3nZ/R8d9)

[Sepúlveda-Vázquez, H. E., C. Villanueva-Mendoza, J. C. Zenteno, V. Villegas-Ruiz, E. Pelcastre-Luna, and G. García-Aguirre. 2014. “Macular Optical Coherence Tomography Findings and GPR143 Mutations in Patients with Ocular Albinism.” *International Ophthalmology* 34 (5): 1075–81. https://doi.org/](http://paperpile.com/b/pec3nZ/npYg)[10.1007/s10792-014-9912-1](http://dx.doi.org/10.1007/s10792-014-9912-1)[.](http://paperpile.com/b/pec3nZ/npYg)

[Sipe, J. D., K. P. McAdam, B. F. Torain, and P. S. Pollock. 1977. “Isolation and Structural Properties of Murine SAA--the Acute Phase Serum Precursor of Amyloid AA.” *Immunological Communications* 6 (1): 1–12. https://doi.org/](http://paperpile.com/b/pec3nZ/n6LC)[10.3109/08820137709055799](http://dx.doi.org/10.3109/08820137709055799)[.](http://paperpile.com/b/pec3nZ/n6LC)

[Somsen, David, Laura Davis-Keppen, Patricia Crotwell, Jason Flanagan, Patrick Munson, and Quinn Stein. 2014. “Congenital Nasal Pyriform Aperture Stenosis and Ocular Albinism Co-Occurring in a Sibship with a Maternally-Inherited 97 Kb Xp22.2 Microdeletion.” *American Journal of Medical Genetics. Part A* 164A (5): 1268–71. https://doi.org/](http://paperpile.com/b/pec3nZ/4ZU7)[10.1002/ajmg.a.36415](http://dx.doi.org/10.1002/ajmg.a.36415)[.](http://paperpile.com/b/pec3nZ/4ZU7)

[Tijmes, N. T., A. B. Bergen, and P. T. De Jong. 1998. “Paucity of Signs in X Linked Ocular Albinism with a 700 Kb Deletion Spanning the OA1 Gene.” *The British Journal of Ophthalmology* 82 (4): 457–58. https://doi.org/](http://paperpile.com/b/pec3nZ/5C9G)[10.1136/bjo.82.4.456b](http://dx.doi.org/10.1136/bjo.82.4.456b)[.](http://paperpile.com/b/pec3nZ/5C9G)

[Trebušak Podkrajšek, Katarina, Branka Stirn Kranjc, Tinka Hovnik, Jernej Kovač, and Tadej Battelino. 2012. “GPR143 Gene Mutation Analysis in Pediatric Patients with Albinism.” *Ophthalmic Genetics* 33 (3): 167–70. https://doi.org/](http://paperpile.com/b/pec3nZ/2uLT)[10.3109/13816810.2011.559651](http://dx.doi.org/10.3109/13816810.2011.559651)[.](http://paperpile.com/b/pec3nZ/2uLT)

[Wang, Y., X. Guo, A. Wei, W. Zhu, W. Li, and S. Lian. 2009. “Identification of a Novel Mutation in a Chinese Family with X-Linked Ocular Albinism.” *European Journal of Ophthalmology* 19 (1): 124–28. https://doi.org/](http://paperpile.com/b/pec3nZ/axYl)[10.1177/112067210901900118](http://dx.doi.org/10.1177/112067210901900118)[.](http://paperpile.com/b/pec3nZ/axYl)

[Xiao, Xueshan, and Qingjiong Zhang. 2009. “Iris Hyperpigmentation in a Chinese Family with Ocular Albinism and the GPR143 Mutation.” *American Journal of Medical Genetics. Part A* 149A (8): 1786–88. https://doi.org/](http://paperpile.com/b/pec3nZ/Ca4D)[10.1002/ajmg.a.32818](http://dx.doi.org/10.1002/ajmg.a.32818)[.](http://paperpile.com/b/pec3nZ/Ca4D)

[Yan, Naihong, Xuan Liao, Su-Ping Cai, Changjun Lan, Yun Wang, Xiaomin Zhou, Yan Yin, Wenhan Yu, and Xuyang Liu. 2012. “A Novel Nonsense Mutation of the GPR143 Gene Identified in a Chinese Pedigree with Ocular Albinism.” *PloS One* 7 (8): e43177. https://doi.org/](http://paperpile.com/b/pec3nZ/OQWa)[10.1371/journal.pone.0043177](http://dx.doi.org/10.1371/journal.pone.0043177)[.](http://paperpile.com/b/pec3nZ/OQWa)

[Zhou, Pingtong, Zhiqiang Wang, Jing Zhang, Landian Hu, and Xiangyin Kong. 2008. “Identification of a Novel GPR143 Deletion in a Chinese Family with X-Linked Congenital Nystagmus.” *Molecular Vision* 14 (May): 1015–19.](http://paperpile.com/b/pec3nZ/klIo) <https://www.ncbi.nlm.nih.gov/pubmed/18523664>[.](http://paperpile.com/b/pec3nZ/klIo)

[Zou, Xuan, Hui Li, Lizhu Yang, Zixi Sun, Zhisheng Yuan, Huajin Li, and Ruifang Sui. 2017. “Molecular Genetic and Clinical Evaluation of Three Chinese Families with X-Linked Ocular Albinism.” *Scientific Reports* 7 (February): 33713. https://doi.org/](http://paperpile.com/b/pec3nZ/3Avv)[10.1038/srep33713](http://dx.doi.org/10.1038/srep33713)[.](http://paperpile.com/b/pec3nZ/3Avv)
